# Anti-NMDAR encephalitis in Crohn’s disease undergoing long-term infliximab treatment: A case report

**DOI:** 10.3389/fimmu.2022.957575

**Published:** 2022-10-31

**Authors:** Shin Ju Oh, Young Nam Kwon, Chang Kyun Lee, Jin San Lee

**Affiliations:** ^1^ Center for Crohn’s and Colitis, Department of Gastroenterology, Kyung Hee University Hospital, Kyung Hee University College of Medicine, Seoul, South Korea; ^2^ Department of Neurology, Seoul National University Hospital, Seoul National University College of Medicine, Seoul, South Korea; ^3^ Department of Neurology, Kyung Hee University Hospital, Kyung Hee University College of Medicine, Seoul, South Korea

**Keywords:** autoimmune encephalitis, anti-N-methyl-D-aspartate, anti-tumor necrosis factor-α, infliximab, Crohn ‘s disease

## Abstract

Infliximab, a chimeric monoclonal antibody against anti-tumor necrosis factor-α (TNF-α), has revolutionized the management of inflammatory bowel disease. However, a recent nested case-control study showed that anti-TNF-α therapy exposure in patients with autoimmune diseases is associated with an increased risk of inflammatory central nervous system (CNS) events. A 27-year-old man diagnosed with Crohn’s disease at 17 years of age was referred to our clinic for suffering with Wernicke’s aphasia and the right-hand weakness over two weeks. Nine years of treatment for Crohn’s disease with infliximab anti-TNF-α therapy was well tolerated. An initial MRI revealed diffuse leptomeningeal enhancement along the bilateral cerebral sulci without any parenchymal abnormalities. Cerebrospinal fluid (CSF) and serum N-methyl-D-aspartate receptor (NMDAR) antibody testing yielded positive results. Anti-NMDAR encephalitis was diagnosed, and the patient was treated with rituximab. A follow-up brain MRI showed new multiple cerebral lesions in the left insular cortex and subcortical white matter of the left frontal and temporal gyri. Approximately 8 months after symptom onset, the CSF and serum NMDAR antibody converted to negative. Twelve months later, the patient fully recovered from anti-NMDAR encephalitis without any neurological deficits and is currently being treated with the anti-interleukin 12/23 agent ustekinumab for Crohn’s disease. This is the first report of not only a patient with infliximab-associated anti-NMDAR encephalitis in Crohn’s disease but also of an inflammatory non-demyelinating CNS event during long-term suppression of TNF-α. Our case highlights the need for clinicians to recognize the possibility of a paradoxical autoimmune response occurring with novel biological therapies.

## Introduction

Anti-tumor necrosis factor-α (TNF-α) therapies have led to a paradigm shift in the management of various immune-mediated inflammatory diseases (IMIDs) such as rheumatoid arthritis, ankylosing spondylitis, and inflammatory bowel disease (IBD), which encompasses Crohn’s disease (CD) and ulcerative colitis (UC). Adverse neurological events associated with anti-TNF-α therapy have been documented in patients with IMIDs, and the most commonly reported associations are demyelinating diseases of the central and peripheral nervous systems. (1) However, a recent nested case-control study has shown that anti-TNF-α therapy exposure in patients with autoimmune diseases is associated with an increased risk of inflammatory demyelinating and non-demyelinating central nervous system (CNS) events. (2) Herein, we report a patient with CD who developed anti-N-methyl-D-aspartate receptor (NMDAR) encephalitis during long-term infliximab (IFX) treatment.

## Case description

A 27-year-old man diagnosed with CD at 17 years of age was referred to our neurology clinic for progressive dysarthria, stuttering, and weakness of the right hand for over 2 weeks. The patient’s CD was initially treated with azathioprine, which yielded satisfactory results. However, owing to the clinical and endoscopic relapse of CD within 2 years of treatment and non-adherence to azathioprine, anti-TNF-α therapy with IFX was added to the treatment plan and azathioprine was discontinued, respectively. IFX monotherapy was well tolerated over 9 years of treatment.

**Figure 1:**
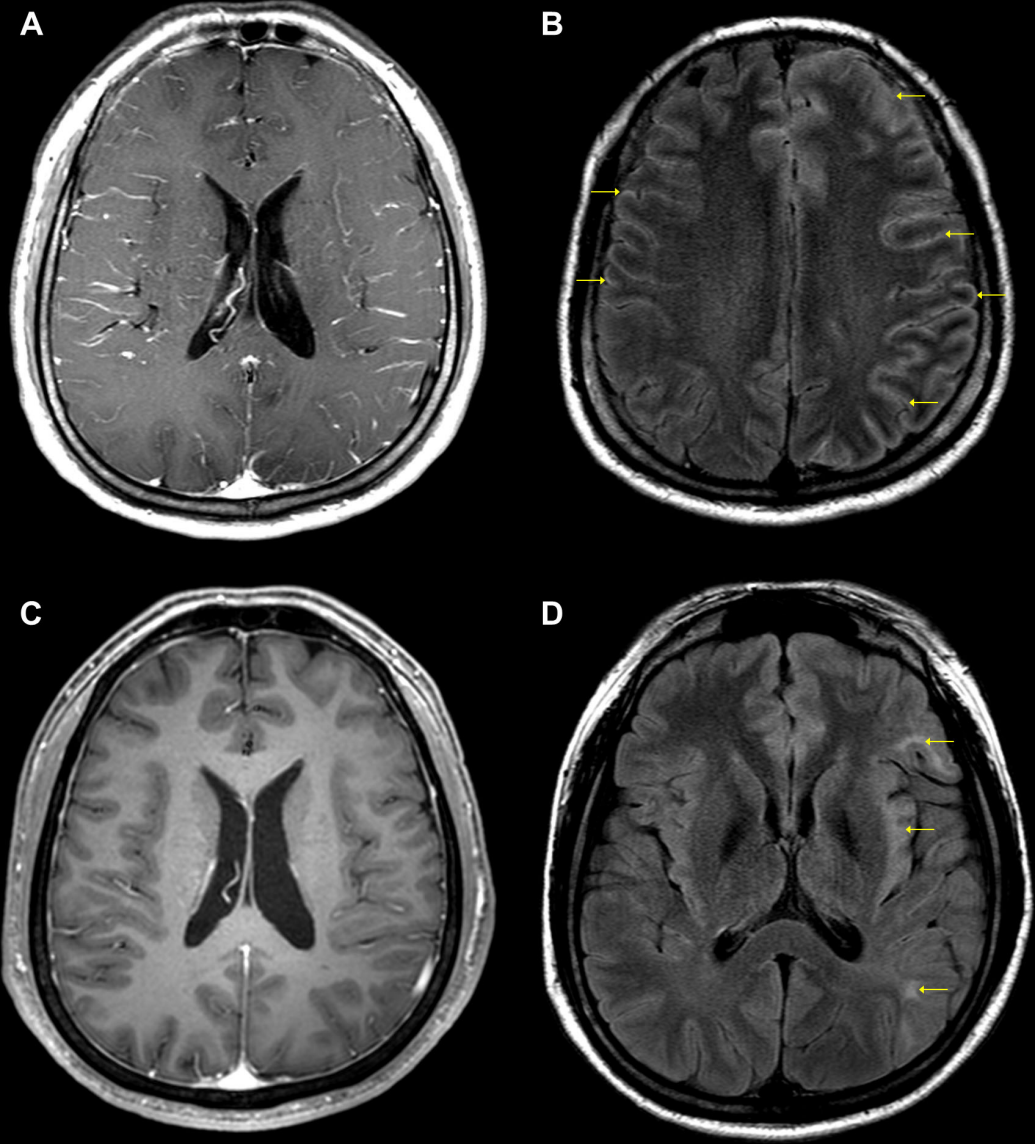
Initial **(A, B)** and follow-up **(C, D)** brain MRIs of the patient. **(A)** Diffuse leptomeningeal enhancement along the bilateral cerebral sulci without any parenchymal abnormalities on post-contrast T1WI. **(B)** Diffuse leptomeningeal enhancement predominantly in the left cerebral sulci (arrows) on contrast-enhanced FLAIR sequence. **(C)** Near-complete resolution of diffuse leptomeningeal enhancement on post-contrast T1WI. **(D)** Multiple cerebral lesions at the left insular cortex and subcortical white matter of the left frontal and temporal gyri (arrows) on FLAIR sequence. TIWI, T1-weighted imaging; FLAIR, fluid-attenuated inversion recovery.

Neurological examination indicated that the cranial nerves were intact, along with normal movements of the extraocular muscles. Motor examination revealed normal muscle strength and tone, except for weakness of the right hand (grade 4 on the Medical Research Council scale). The Babinski sign was not elicited, and sensory examination results were normal for all modalities. However, the patient exhibited profound deficits in comprehension on language examination. The patient’s fluency and repetition were relatively preserved, suggesting the occurrence of Wernicke’s aphasia. An initial brain MRI revealed diffuse leptomeningeal enhancement along the bilateral cerebral sulci without any parenchymal abnormalities on post-contrast T1-weighted imaging (**Figure 1A**). This enhancement pattern was more prominent in the left cerebral sulci on the contrast-enhanced fluid-attenuated inversion recovery (FLAIR) sequence (**Figure 1B**). In addition, electroencephalography demonstrated continuous slow activity over the left frontocentral region, without epileptiform discharges.

Based on the patient’s neurological deficits and the neuroimaging findings, encephalitis was suspected, and an additional diagnostic workup was performed. Extensive serological and hematological tests, including a complete blood count, blood chemistry, bacterial and viral infection markers, antinuclear antibodies, extractable nuclear antigen antibodies, antiphospholipid and lupus anticoagulant antibodies, and hormonal panels, were all normal. Cerebrospinal fluid (CSF) analysis revealed lymphocytic pleocytosis (60 cells/µL), with normal protein and glucose levels. As CSF testing for viral and CNS demyelinating diseases yielded negative results, we suspected autoimmune encephalitis and hence, discontinued IFX. Although the patient was administered 1,000 mg of methylprednisolone intravenously (IV) daily for 5 days, followed by IV immunoglobulin at 0.4 g/kg daily for 5 days, there was no significant clinical improvement. On the third day of immunoglobulin treatment, the patient exhibited a generalized tonic-clonic epileptic seizure with forced eye deviation to the right, followed by right-side head turning for 2 min. Antiseizure medication (levetiracetam) was administered immediately, and the seizure stopped.

On hospital day 12, bacterial and fungal CSF cultures were negative; however, among various neuronal autoantibodies, including anti-myelin oligodendrocyte glycoprotein (MOG) antibody analyzed using a cell-based immunocytochemistry assay kit (Euroimmun Ag, Lübeck, Germany), only CSF and serum NMDAR antibody testing yielded positive results (1:40 titer). Anti-NMDAR encephalitis was diagnosed, and the patient was treated with IV rituximab (375 mg/m2; 650 mg per week) for 4 weeks. The symptoms started to improve on the second day after the first dose of rituximab. On hospital day 14, only mild anomia and stuttering remained, suggesting a rapid recovery from the initial Wernicke’s aphasia. A follow-up brain MRI was performed after the last dose of rituximab, which indicated near-complete resolution of the diffuse leptomeningeal enhancement (**Figure 1C**). However, new multiple cerebral lesions with hyperintensities appeared on the FLAIR sequence in the left insular cortex and subcortical white matter of the left frontal and temporal gyri (**Figure 1D**). On hospital day 39, repeated testing for CSF and serum NMDAR antibodies still yielded positive result, and the antibody titer dropped below 1:10. No significant CSF pleocytosis (3 cells/µL) was noted. The patient was discharged from our hospital after recovering enough to be able to perform daily activities without neurological deficits. The third follow-up brain MRI performed approximately 8 months after symptom onset showed no change from the second neuroimaging findings; however, the NMDAR antibodies were negative. Twelve months later, the patient fully recovered from anti-NMDAR encephalitis without any neurological deficits and is currently being treated with the anti-interleukin 12/23 agent ustekinumab for CD.

## Discussion

IFX, a chimeric monoclonal antibody against TNF-α, has revolutionized IBD management since its first clinical use in a pediatric patient with refractory CD in 1993. (3) Interestingly, widespread use of anti-TNF therapies in IMIDs can paradoxically increase the risk of immune-mediated adverse effects of the drugs. To the best of our knowledge, this is the first report of not only a patient with IFX-associated anti-NMDAR encephalitis in CD but also of an inflammatory non-demyelinating CNS event during long-term suppression of TNF-α by IFX treatment. There was no evidence of tumors or prior herpes simplex encephalitis – two confirmed triggers of anti-NMDAR encephalitis (4) – in our patient. We found two recent reports of anti-NMDAR encephalitis associated with anti-TNF therapy for IBD. (5, 6) The symptoms of encephalitis in both cases developed 6 months after initiating adalimumab. We also found a case of anti-NMDAR encephalitis that occurred during treatment with adalimumab for psoriasis for 5 months. (7) However, those in the present case occurred at 9 years despite the good therapeutic adherence to IFX in the patient, without any reported adverse events during the long treatment period. Although there was no temporal association between disease onset and anti-TNF-α therapy in our case, we speculate that long-term treatment with IFX could also be associated with the development of anti-NMDAR encephalitis, based on a recent report of anti-MOG-associated encephalitis in a patient with UC who received IFX for 10 years. (8)

This report is consistent with our case in that autoimmune encephalitis similarly occurred during long-term use of the same drug in patients with IBD. However, there were differences in the neuroimaging findings between the two cases. The previous report documented the first case of unilateral cortical FLAIR-hyperintense Lesions in Anti-MOG-associated Encephalitis with Seizures (FLAMES) while on anti-TNF-α therapy. FLAMES is a recently characterized manifestation of MOG antibody-associated disorder that is defined as unilateral cortical FLAIR hyperintensity without involvement of the adjacent white matter on MRI. (9) In patients with anti-NMDAR encephalitis, normal brain MRI findings are observed in half of the patients, and lesions in the hippocampus are the most common abnormal MRI findings. (10) The initial brain MRI in our case revealed diffuse leptomeningeal enhancement along the bilateral cerebral sulci without any cortical or subcortical FLAIR hyperintensity, consistent with a recently reported case. (11) Follow-up brain MRI in our case showed that the unilateral subcortical white matter lesions were still present after 8 months, whereas in a previously reported case, it showed near-resolution of unilateral cortical lesions. Studies on patients with anti-NMDAR encephalitis have reported that approximately 5% of cases develop radiological evidence of a CNS demyelinating disorder, and these radiological features can precede, occur simultaneously, or develop after anti-NMDAR encephalitis in a setting where anti-MOG antibodies coexist. (12, 13) Although no anti-MOG antibodies were detected in our patient, lesions were found on subsequent MRI. Therefore, further investigations are required to determine whether additional neuronal autoantibodies coexist in the patient.

The mechanisms by which long-term IFX treatment leads to anti-NMDAR encephalitis remain unknown. The proposed mechanisms of inflammatory CNS events associated with anti-TNF-α therapy include autoimmune dysregulation accompanied by abnormal B-cell activation in the CNS by autoreactive T cells. (14, 15) Long-term TNF-α inhibition can result in upregulation of TNF expression, and since TNF-α inhibitors cannot cross the blood-brain barrier, a paradoxical increase in TNF within the CNS may occur. (2, 14) In addition, in complex cytokine environments such as the intestinal mucosa, TNF-α inhibition can result in local aberrant priming and expansion of B-cell autoreactivity. (5) The fact that B-cell depletion therapy using rituximab improved patient symptoms in several reports, including this case, provides support for these mechanisms. Given the increasing incidence of inflammatory CNS events related to anti-TNF-α therapy, (2) the immunopathogenic mechanisms underlying autoimmune encephalitis warrant further investigation.

However, since our case lacked a temporal association between disease onset and anti-TNF-α therapy, we could not rule out the possibility that anti-TNF-α therapy-associated aseptic meningitis, CNS demyelination, and anti-NMDAR antibody positivity simultaneously occurred in this patient. A previous report showed that aseptic meningitis, including the development of leptomeningeal enhancement, can occur in patients with rheumatoid arthritis and ankylosing spondylitis during long-term anti-TNF-α therapy; however, the incidence is extremely low (4/718, 0.56%). (16) The onset of clinical symptoms developed after a mean of 5 years of drug exposure. Moreover, it is increasingly being recognized that CNS demyelinating events and aseptic meningitis with leptomeningeal enhancement can be associated with the use of anti-TNF-α therapy. (2) Although Wernicke’s aphasia and focal to bilateral tonic-clonic seizure in the present case may be part of the presentation of anti-NMDAR encephalitis, the other features (focal limb weakness, the imaging findings of leptomeningeal enhancement, and delayed appearance of multiple FLAIR hyperintensities) are not indications of anti-NMDAR encephalitis. These findings, along with anti-NMDAR encephalitis, could be partly supported by the possibility of co-occurrence of aseptic meningitis and CNS demyelination.

Here, we demonstrate the occurrence of anti-NMDAR encephalitis after long-term exposure to anti-TNF-α therapy in a young man with longstanding CD. Our case highlights that clinicians should recognize the possibility of a paradoxical autoimmune response occurring with novel biological therapies and consider these drugs as potential etiologies when new neurological impairments develop in patients with previously diagnosed autoimmune or rheumatological diseases.

## Data availability statement

The original contributions presented in the study are included in the article/supplementary material. Further inquiries can be directed to the corresponding author.

## Ethics statement

The studies involving human participants were reviewed and approved by Institutional Review Board of Kyung Hee University Hospital. The patients/participants provided their written informed consent to participate in this study.

## Author contributions

Conception and design of the study: CL and JL. Acquisition of data: SO, CL, and JL. Analysis and interpretation of the data: SO, YK, CL, and JL. Drafting and revising the manuscript for content: SO, YK, CL, and JL. Final approval of the manuscript: CL and JL. All authors contributed to the article and approved the submitted version.

## Funding

This work was supported by the National Research Foundation of Korea (NRF) grant funded by the Korea government (Ministry of Science and ICT) (NRF-2021R1C1C1012429) and the Korea Health Industry Development Institute (KHIDI, grant no. HU21C0098).

## Conflict of interest

The authors declare that the research was conducted in the absence of any commercial or financial relationships that could be construed as a potential conflict of interest.

## Publisher’s note

All claims expressed in this article are solely those of the authors and do not necessarily represent those of their affiliated organizations, or those of the publisher, the editors and the reviewers. Any product that may be evaluated in this article, or claim that may be made by its manufacturer, is not guaranteed or endorsed by the publisher.

## References

[1] Deepak P Stobaugh DJ Sherid M Sifuentes H Ehrenpreis ED. Neurological events with tumour necrosis factor alpha inhibitors reported to the food and drug administration adverse event reporting system. Aliment Pharmacol Ther (2013) 38:388–96. doi: 10.1111/apt.12385 23802849

[2] Kunchok A Aksamit AJ Jr. Davis,JM 3rd Kantarci,OH Keegan BM Pittock SJ. Association between tumor necrosis factor inhibitor exposure and inflammatory central nervous system events. JAMA Neurol (2020) 77:937–46. doi: 10.1001/jamaneurol.2020.1162 PMC7235930 32421186

[3] Derkx B Taminiau J Radema S Stronkhorst A Wortel C Tytgat G. Tumour-necrosis-factor antibody treatment in crohn’s disease. Lancet (1993) 342:173–4. doi: 10.1016/0140-6736(93)91375-V 8101267

[4] Dalmau J Armangué T Planagumà J Radosevic M Mannara F Leypoldt F. An update on anti-NMDA receptor encephalitis for neurologists and psychiatrists: mechanisms and models. Lancet Neurol (2019) 18:1045–57. doi: 10.1016/S1474-4422(19)30244-3 31326280

[5] Noble GP Lancaster E. Anti-NMDAR encephalitis in a patient with crohn disease receiving adalimumab. Neurol Neuroimmunol Neuroinflamm (2018) 5:e476. doi: 10.1212/NXI.0000000000000476 29988714 PMC6029580

[6] Fernández Alvarez P Maldonado Pérez B Castro Laria L Argüelles-Arias F. Autoimmune encephalitis during treatment with adalimumab: A case report in crohn’s disease. Inflammation Bowel Dis (2021) 27:e40–1. doi: 10.1093/ibd/izaa302 33210723

[7] Demarez B Amatore F Lagarde S Bruder N Grob JJ Richard MA. Anti-N-methyl-D-aspartate receptor encephalitis during treatment with adalimumab for psoriasis. J Eur Acad Dermatol Venereol (2020) 34:e591–3. doi: 10.1111/jdv.16456 32279373

[8] Chang YC Sharma M Budhram A. Unilateral cortical FLAIR-hyperintense lesion in anti-MOG-associated encephalitis with seizures (FLAMES) on TNF inhibitor therapy. J Neurol Neurosurg Psychiatry (2021). doi: 10.53347/rID-89390 33785577

[9] Budhram A Mirian A Le C Hosseini-Moghaddam SM Sharma M Nicolle MW. Unilateral cortical FLAIR-hyperintense lesions in anti-MOG-associated encephalitis with seizures (FLAMES): characterization of a distinct clinico-radiographic syndrome. J Neurol (2019) 266:2481–7. doi: 10.1007/s00415-019-09440-8 31243540

[10] Zhang T Duan Y Ye J Xu W Shu N Wang C. Brain MRI characteristics of patients with anti-N-Methyl-D-Aspartate receptor encephalitis and their associations with 2-year clinical outcome. AJNR Am J Neuroradiol (2018) 39:824–9. doi: 10.3174/ajnr.A5593 PMC7410660 29567651

[11] Park JK Lee EJ Kim KK. Isolated leptomeningeal enhancement in anti-N-Methyl d-aspartate receptor encephalitis: The diagnostic value of contrast-enhanced fluid-attenuated inversion recovery imaging. J Korean Soc Radiol (2022) 83:945–50. doi: 10.3348/jksr.2021.0098 PMC9550631 36238909

[12] Hacohen Y Absoud M Hemingway C Jacobson L Lin JP Pike M. NMDA receptor antibodies associated with distinct white matter syndromes. Neurol Neuroimmunol Neuroinflamm (2014) 1:e2. doi: 10.1212/NXI.0000000000000002 25340058 PMC4202680

[13] Titulaer MJ Höftberger R Iizuka T Leypoldt F Mccracken L Cellucci T. Overlapping demyelinating syndromes and anti–N-methyl-D-aspartate receptor encephalitis. Ann Neurol (2014) 75:411–28. doi: 10.1002/ana.24117 PMC4016175 24700511

[14] Robinson WH Genovese MC Moreland LW. Demyelinating and neurologic events reported in association with tumor necrosis factor alpha antagonism: by what mechanisms could tumor necrosis factor alpha antagonists improve rheumatoid arthritis but exacerbate multiple sclerosis? Arthritis Rheum (2001) 44:1977–83. doi: 10.1002/1529-0131(200109)44:9<1977::AID-ART345>3.0.CO;2-6 11592357

[15] Jain RW Yong VW. B cells in central nervous system disease: diversity, locations and pathophysiology. Nat Rev Immunol (2021) 22 (8): 513–24. doi: 10.1038/s41577-021-00652-6 34903877 PMC8667979

[16] Cavazzana I Taraborelli M Fredi M Tincani A Franceschini F. Aseptic meningitis occurring during anti-TNF-alpha therapy in rheumatoid arthritis and ankylosing spondylitis. Clin Exp Rheumatol (2014) 32:732–4. Available at: https://www.clinexprheumatol.org/abstract.asp?a=8056 25198168

